# Five new species of Aspidiotini (Hemiptera, Diaspididae, Aspidiotinae) from Argentina, with a key to Argentine species

**DOI:** 10.3897/zookeys.948.54618

**Published:** 2020-07-13

**Authors:** Scott A. Schneider, Lucia E. Claps, Jiufeng Wei, Roxanna D. Normark, Benjamin B. Normark

**Affiliations:** 1 USDA, Agricultural Research Service, Henry A. Wallace Beltsville Agricultural Research Center, Systematic Entomology Laboratory, Building 005 - Room 004, 10300 Baltimore Avenue, Beltsville, MD 20705, USA; 2 Universidad Nacional de Tucumán. Facultad de Ciencias Naturales e Instituto Miguel Lillo, Instituto Superior de Entomología “Dr. Abraham Willink”, Batalla de Ayacucho 491, T4000 San Miguel de Tucumán, Tucumán, Argentina; 3 College of Agriculture, Shanxi Agricultural University, Taigu, Shanxi, 030801, China; 4 Department of Biology, University of Massachusetts, 221 Morrill Science Center III 611 North Pleasant Street, Amherst, MA 01003, USA; 5 Graduate Program in Organismic and Evolutionary Biology, University of Massachusetts, 204C French Hall, 230 Stockbridge Road Amherst, MA 01003, USA

**Keywords:** armored scale insects, *Chortinaspis*, *Clavaspis*, Coccoidea, Coccomorpha, *Hemiberlesia*, *Melanaspis*, taxonomy

## Abstract

Five new species of armored scale insect from Argentina are described and illustrated based upon morphological and molecular evidence from adult females: *Chortinaspisjujuyensis***sp. nov.**, *Clavaspispatagonensis***sp. nov.**, *Hemiberlesiaozolita***sp. nov.**, *Melanaspislilloi***sp. nov.**, and *Melanaspistargionoides***sp. nov.** The genera *Chortinaspis* and *Melanaspis* are recorded for the first time from this country. An identification key to all recorded species from tribe Aspidiotini occurring in Argentina is provided.

## Introduction

Armored scale insects are ubiquitous, highly invasive herbivores that often become pests on trees, shrubs, and ornamentals (Miller and Davidson 1990; 2005; Normark et al. 2019). They comprise the largest family of scale insects (Hemiptera, Coccomorpha, Diaspididae) with over 2600 species in 422 genera (García Morales et al. 2016). About one-quarter of armored scales belong to the tribe Aspidiotini Westwood, a particularly pestiferous group containing numerous cosmopolitan species (Miller and Davidson 1990; Schneider et al. 2019). At present the tribe comprises over 720 species in 88 genera; however, recent phylogenetic evidence has revealed rampant artificiality among these genera (Schneider et al. 2018), and their classification is in need of extensive revision.

This article describes five new species of Aspidiotini from Argentina. Generic designations are made following morphology and the best available evidence from molecular studies (Schneider et al. 2018; Normark et al. 2019), keeping in mind the ultimate goal to identify genera of Aspidiotini that delimit natural groups of species. This article also provides an identification key to the species of Aspidiotini recorded from Argentina (García Morales et al. 2016, last accessed 21.V.2020). This work adds to our knowledge of armored scale diversity in this region, for which a foundation has largely been established by Claps, Wolff, and colleagues (Claps and Terán 2001; Claps et al. 2001a; b; Claps and Wolff 2003; 2014; Granara de Willink and Claps 2003; and additional works).

Sixteen genera from tribe Aspidiotini are now recorded in Argentina (García Morales et al. 2016), including: *Acutaspis* Ferris, 1941; *Aonidiella* Berlese & Leonardi, 1896; *Aspidiotus* Bouché, 1833; *Chortinaspis* Ferris, 1938 (new record); *Chrysomphalus* Ashmead, 1880; *Clavaspis* MacGillivray, 1921; *Comstockaspis* MacGillivray, 1921; *Crenulaspidiotus* MacGillivray, 1921; *Diaspidiotus* Berlese & Leonardi, 1896; *Hemiberlesia* Cockerell, 1897; *Lindingaspis* MacGillivray, 1921; *Melanaspis* Cockerell, 1897 (new record); *Mycetaspis* Cockerell, 1897; *Oceanaspidiotus* Takagi, 1984; *Pseudischnaspis* Hempel, 1900; and *Targionia* Signoret, 1869.

## Materials and methods

Specimens were preserved in 100% ethanol and stored at -20 °C before being slide-mounted. Specimens were slide-mounted according to the joint DNA-morphology preparation protocol described in Normark et al. (2019), except that some specimens were prepared according to an earlier set of protocols. In the earlier protocols some specimens were ground to powder for DNA preparation and others from the same series were transferred directly to 10% KOH for mounting on microscope slides.

Morphological terminology conforms to descriptions and illustrations provided by Schneider et al. (2019). Measurements were made on a Zeiss Axio Imager.M2 (Carl Zeiss Microscopy, LLC, White Plains, NY, USA) microscope with the aid of an AxioCam and AxioVision software. Illustrations were made with the aid of a camera lucida. Slide-mounted specimens were examined by the authors under phase contrast and Differential Interference Contrast microscopy.

Depositories are abbreviated as follows: IFML, Instituto Fundación Miguel Lillo, Tucumán, Argentina; USNM, United States National Museum, scale insect collection at Agricultural Research Service, Beltsville, Maryland, USA; UMEC, University of Massachusetts Entomology Collection, Amherst, Massachusetts, USA.

## Taxonomy

### Chortinaspis jujuyensis

Taxon classificationAnimaliaHemipteraDiaspididae

Schneider, Claps, Wei, Normark & Normark sp. nov.

DDE4F300-800C-5E90-A8EE-6423D58B17E5

http://zoobank.org/33040435-CC00-4B65-A5F8-687A02E4BCA8

Figs 1 , 2

#### Material examined.

***Holotype***: Argentina • 1 adult female; Jujuy, Humahuaca, Ruta 9, entrada a Iruya; 22.997S, 65.369W; 12.II.2002; L. E. Claps, P. Zamudio, L. Díaz-Briz, and P. Cabrera leg.; IFML, L. E. Claps catalog #12-02, # 1089 (D0265G). ***Paratypes***: Argentina • 2 adult females; same slide and data as holotype; IFML (D0265G) • 1 adult female; same data as holotype; IFML (D0265H) • 1 adult female; same data as holotype; IFML (D0265J) • 1 adult female; same data as holotype; IFML (D0265K) • 3 adult females; same data as holotype; UMEC (D0265I) • 1 adult female; same data as holotype; USNM (D0265L) • 1 adult female; same data as holotype; USNM (D0265M).

#### Description

(*N* = 11). Adult female presumed to secrete scale cover, not pupillarial. Appearance in life not recorded. Slide-mounted adult female 730–1110 (holotype 860, median 860) μm long, 590–800 (holotype 680, median 680) μm wide; broadest near mesothorax and metathorax. Body outline nearly oval. Derm membranous except for pygidium. Antennae simple, each with one thick, flagellate seta; distance between antennae 120–150 (median 130) μm. Without disc pores near anterior or posterior spiracles. ***Lobes***: Pygidium with 2 pairs of well-developed sclerotized lobes extending from pygidial margin. Median lobes (L1) prominent and broad, roughly rectangular in shape with ragged edges; each lobe with basal scleroses nearly equal in length to L1, broad basally and tapering anteriorly; L1 separated by interlobular space about 1/4 width of L1; second lobes (L2) about 1/2 width of L1, smoothly rounded apically, without notches, L3 and L4 absent. ***Paraphyses***: Absent. ***Plates***: 1 pair of simple plates between L1, with shallow bifurcations, not deeply fringed, slightly longer than L1; 2 plates present in first space between L1 and L2, the plate immediately anterior to L1 simple and roughly triangular, the other roughly rectangular and apically fringed, both longer than L1; 2 plates anterior to L2, variously fringed, ranging from simple to fimbriate; plates absent beyond setae marking position of L3. ***Ducts***: Dorsal pygidial macroducts of 1-barred type, long and slender, duct filaments about 6–8 times as long as width of orifices; 1 macroduct between L1 (rarely absent), extending beyond posterior margin of anal opening, 40–51 (median 45) μm in length; 5–9 clustered macroducts arising from first space between L1 and L2, 14–27 on abdominal segment VI, in elongate cluster arising from second space and widening anteriorly; 18–38 ducts on abdominal segment V, in irregular, elongate cluster arising from third space and widening anteriorly; 38–66 (median 48.5) macroducts on each side of pygidium in total. Submarginal cluster of 4–17 (median 11) macroducts present on abdominal segment IV; few marginal macroducts present on each of abdominal segments I–III and metathorax. Dorsal submedial groups of microducts present on each of abdominal segments I–III. Small clusters of ventral submarginal microducts present on abdominal segments II–VI. ***Anal opening***: Small and slightly oval, 11–17 (median 14) μm in diameter, positioned 2.2–3.3 (median 2.3) anal lengths from base of L1, located in posterior third of pygidium. ***Perivulvarpores***: Absent.

**Figure 1.**
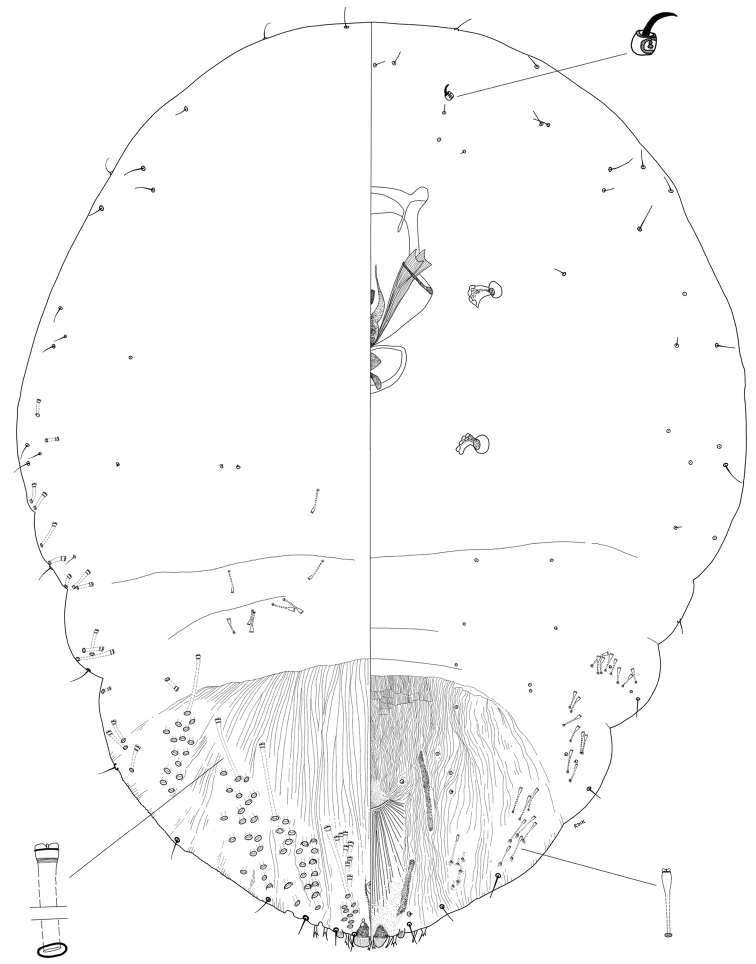
*Chortinaspisjujuyensis* sp. nov. Adult female, full body view, illustrated from the holotype (D0265G).

#### DNA sequences.

Several DNA sequences of *Chortinaspisjujuyensis* sp. nov. have been published, including fragments of 4 loci. None are from the holotype or paratypes, but all are from specimens collected in the same collecting event with the same data. Specimen D0265A was ground to powder during DNA preparation. Specimens D0265E and D0265F are mounted on microscope slides but are in poor condition; they are identifiable as *C.jujuyensis* sp. nov. but were not suitable for reliable measurements and therefore were not designated as paratypes. The sequenced loci and corresponding GenBank accession numbers are: the large ribosomal subunit (28S; D0265A, DQ145314.2; D0265F, MH933984.1), elongation factor 1-alpha (EF-1α; D0265A, DQ145426.1; D0265E, MH915708.1; D0265F, MH915709.1), carbamoylphosphate synthetase (CAD; D0265E, MH915983.1; D0265F, MH915984.1), and cytochrome oxidase I and II (COI-II; D0265, GQ424990.1; D0265E, MH916219.1 & MH916391.1; D0265F, MH916220.1 & MH916392.1). The small ribosomal subunit (16S) sequences of the primary bacterial endosymbiont, *Uzinuradiaspidicola*, of *C.jujuyensis* sp. nov. has also been published: GQ424853.1.

**Figure 2.**
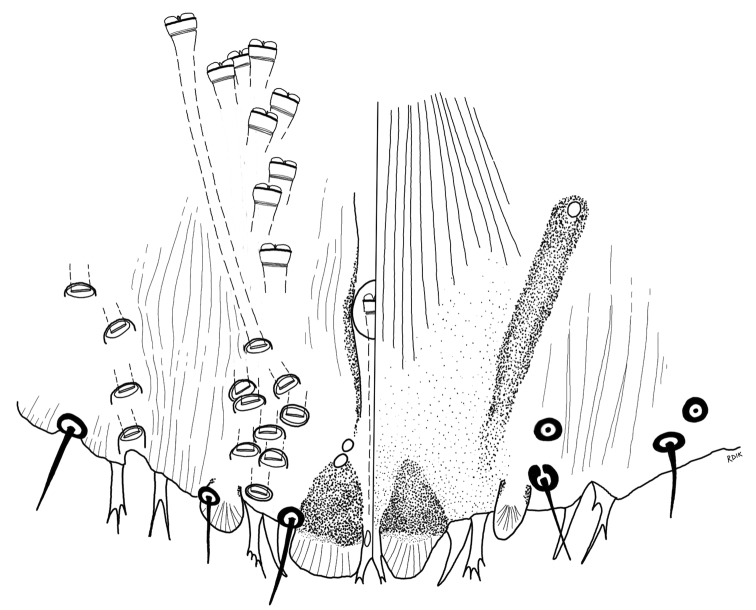
*Chortinaspisjujuyensis* sp. nov. Adult female, expanded view of pygidium, illustrated from the holotype (D0265G).

#### Informal synonyms.

Specimens of *C.jujuyensis* sp. nov. have appeared in several published phylogenetic trees, and have been referred to variously as “Diaspidiotus sp undesc #2” (Morse and Normark 2006; Rugman-Jones et al. 2010), “Diaspidiotus sp. undesc.” (Gruwell et al. 2009), “Diaspidiotus sp” (Andersen et al. 2010), and “Chortinaspis ud0265” (Schneider et al. 2018; Normark et al. 2019).

#### Remarks.

*Chortinaspisjujuyensis* sp. nov. shares similarities with *C.graminella* (Cockerell) and *C.frankliniana* Ferris. The median lobes of *C.jujuyensis* sp. nov. are apically truncate or nearly rectangular in shape like those of *C.graminella* and have rough apical edges like those of *C.frankliniana*. But *C.jujuyensis* sp. nov. can be distinguished from both species by its narrow, smooth second lobes, in contrast to the broadly truncate and notched second lobes seen in the other two species. It differs from *C.chortina* (Ferris) in that it lacks any plates anterior to the position of the third lobes.

#### Host plant.

Not recorded.

#### Etymology.

The specific epithet is an adjective formed from the name Jujuy, the province in which it was found + the suffix -*ensis*, meaning of or from a place.

#### Distribution.

Argentina (Jujuy).

### Clavaspis patagonensis

Taxon classificationAnimaliaHemipteraDiaspididae

Schneider, Claps, Wei, Normark & Normark sp. nov.

AEEA2993-B364-506A-BD0A-4E951307D422

http://zoobank.org/B7FD9835-4FAE-4CE0-8B8A-1E11DB6A4705

Figs 3 , 4

#### Material examined.

***Holotype***: Argentina • 1 adult female; Neuquén, PN Lanin, Pucará; 40.15S, 71.63W; 28.XI.2001; L. Claps and L. Díaz Briz leg.; IFML, L. E. Claps catalog # 16-01, #1090 (D0274E). ***Paratypes***: Argentina • 1 adult female; same slide as holotype; IFML (D0274E) • 1 adult female; same data as holotype; UMEC (D0274B) • 1 adult female; same data as holotype; USNM (D0274A).

#### Description

(*N* = 4). Adult female presumed to secrete scale cover, not pupillarial. Appearance in life not recorded. Slide-mounted adult female 850–1240 (holotype 1240) μm long, 780–1000 (holotype 1000) μm wide; broadest near mesothorax. Body outline turbinate. Derm membranous throughout at maturity except for light pygidial sclerotization. Antennae simple, each with one spine-like seta. Distance between antennae 150–185 μm. Without disc pores associated with anterior or posterior spiracles. ***Lobes***: Only L1 well developed and sclerotized, slightly wider than long, inner margins parallel or slightly converging, with 0–1 medial notch and 1–2 lateral notches; median lobes separated by space 1/5 their width; L2 and L3 absent in typical form, one specimen with single poorly formed L2 present in type series. ***Paraphyses***: With 1 pair of paraphysis-like pyriform sclerotizations between L1; interlobular spaces between L1 and L2 and between L2 and L3 each with 2 clavate paraphyses, inner paraphysis slightly larger than outer paraphysis of each pair; paraphyses arising from lateral angle of L1 only slightly swollen at anterior end and directed away from meson. ***Plates***: Difficult to observe; apparently 1 or 2 present between L1 and L2, 2 present between L2 and L3, 0–3 beyond L3, all roughly rectangular with minor fringing at apex, about as long as L1. ***Ducts***: Dorsal pygidial macroducts of 1-barred type; one macroduct present between median lobes with duct exceeding beyond posterior margin of anal opening; with 2–3 macroducts arising from first interlobular space; roughly single-file row of 7–8 macroducts arising from second interlobular space; 8–13 in marginal and submarginal areas of abdominal segment V, arising from third interlobular space. Few pre-pygidial macroducts on marginal line from mesothorax to abdominal segment III, 1–3 per segment on each side, shorter than pygidial macroducts; 1–2 submarginal macroducts present on each side of abdominal segment IV; small sets of 1–4 short submedial macroducts present on each side of abdominal segments I–IV. Ventral marginal or submarginal microducts present in small groups on each segment from prothorax to abdominal segment VI. ***Anal opening***: Positioned in posterior third of pygidium, 12–14 μm in diameter, positioned about 2 anal lengths from base of L1. ***Perivulvarpores***: Divided into 4 or sometimes 5 groups, 2–7 in each anterolateral, 3–4 in each posterolateral group, and 0–2 in anterior group; 12–21 pores in total.

#### DNA sequences.

DNA sequences of *Clavaspispatagonensis* sp. nov. have been published, all from one of the paratypes (D0274B): 28S, GenBank accession number KY218988.1; EF-1α, MH915713.1 and KY221285.1; COIII, MH916221.1 and KY220694.1; 16S of primary endosymbiont (*Uzinuradiaspidicola*), KY220094.1.

**Figure 3.**
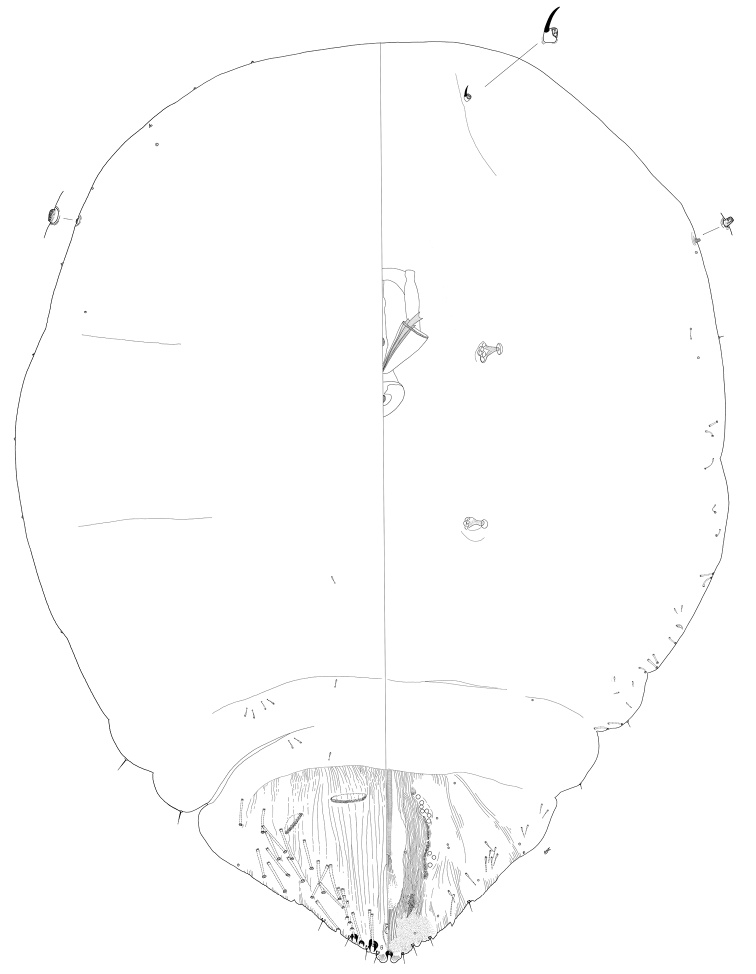
*Clavaspispatagonensis* sp. nov. Adult female, full body view, illustrated from the holotype (D0274E).

#### Informal synonyms.

A specimen from the type series (D0274B) has appeared in published molecular-phylogenetic analyses, designated as “Clavaspis undescr” (Schneider et al. 2018) and “Clavaspis ud0274” (Normark et al. 2019).

#### Remarks.

The traditional morphology-based assignment for this species would be in the genus *Diaspidiotus*, but recent molecular-phylogenetic studies have shown that *Diaspidiotus* is radically non-monophyletic and that the true affinities of this species lie with the genus *Clavaspis* (Schneider et al. 2018). The morphological character traditionally used to distinguish between these genera is the shape of paraphyses arising from the lateral angles of median lobes. In typical *Clavaspis* species, these paraphyses are swollen at the anterior end and directed toward the midline of the body or they have a detached knob giving them a mushroom-like appearance (Ferris 1938). In *C.patagonensis* sp. nov., the paraphyses are slightly swollen at the anterior end but they are pointing away from the midline, similar in appearance to those found in species of *Diaspidiotus* and other near relatives, like *Hemiberlesia*.

**Figure 4.**
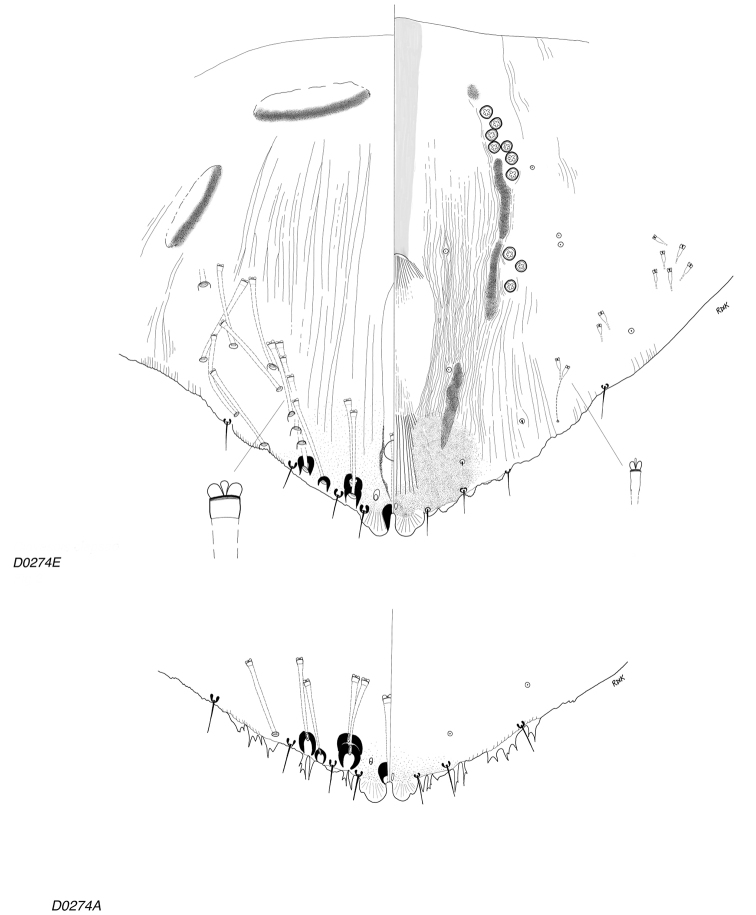
*Clavaspispatagonensis* sp. nov. Adult female, expanded view of pygidium, illustrated from the holotype (D0274E) and a paratype (D0274A), reflecting variation in the degree of visibility of the plates.

Adult females of *C.patagonensis* sp. nov. are nearly identical in appearance to *C.covilleae* (Ferris), but the species are separated on the phylogeny by several other members of *Clavaspis*. The two can be distinguished based on the shape of paraphyses arising from the lateral angles of median lobes and the distribution of macroducts. *Clavaspispatagonensis* sp. nov. has fairly narrow paraphyses and possesses one or two dorsal submarginal macroducts on abdominal segment IV. *Clavaspiscovilleae* has broadly swollen paraphyses, typical of *Clavaspis*, and lacks any submarginal macroducts on abdominal segment IV. The new species could also be easily confused with *Diaspidiotusosborni* (Newell & Cockerell). In this case, *C.patagonensis* sp. nov. can be distinguished by possessing submarginal macroducts on IV, having more than one marginal macroduct on at least one pre-pygidial segment, lacking dorsal submarginal microducts on pre-pygidial segments, and having a prosoma that remains membranous in mature adult females. In contrast, *D.osborni* lacks submarginal macroducts on IV, typically has one marginal macroduct per pre-pygidial segment, has small groups of dorsal submarginal microducts on pre-pygidial segments, and the prosoma becomes sclerotized in mature adult females.

#### Host plant.

*Embothriumcoccineum* J. R. Forst. & G. Forst. (Proteaceae)

#### Etymology.

The specific epithet is an adjective formed from the name Patagonia, the region in which it was found + the suffix -*ensis*, meaning of or from a place.

#### Distribution.

Argentina (Neuquén).

### Hemiberlesia ozolita

Taxon classificationAnimaliaHemipteraDiaspididae

Schneider, Claps, Wei, Normark & Normark sp. nov.

03243AED-DF51-59B1-B660-4182E4EE2FF9

http://zoobank.org/680825CA-8604-4D3C-9717-39B1AD53A423

Figs 5 , 6

#### Material examined.

***Holotype***: Argentina • 1 adult female; Jujuy, Humahuaca, camino a Aparzo; 23.20S, 65.10W; 14.II.2002; L. E. Claps, P. Zamudio, L. Díaz-Briz, P. Cabrera leg.; IFML, L. E. Claps catalog #22-02, #1091 (D0288D). ***Paratypes***: Argentina • 1 adult female; same slide and same data as holotype; IFML (D0288D) • 1 adult female; same data as holotype; USNM (D0288C) • 1 adult female; same data as holotype; USNM (D0288G) • 1 adult female; same data as holotype; UMEC (D0288F) • 1 adult female; same data as holotype; UMEC (D0288H) • 1 adult female; same data as holotype; UMEC (D0288I) • 1 adult female; same data as holotype; UMEC (D0288J).

#### Description

(*N* = 8). Adult female presumed to secrete scale cover, not pupillarial. Appearance in life not recorded. Slide-mounted adult female 770–1050 (median 910, holotype 990) μm long, 660–810 (median 780, holotype 810) μm wide; broadest at mesothorax. Body outline nearly circular; derm of prosoma becoming slightly sclerotized at full maturity (body length > 1mm), otherwise derm membranous except for pygidium. Antennae simple, each with one spine-like seta; distance between antennae 120–180 (median 140) μm. Without disc pores near anterior or posterior spiracles. ***Lobes***: Only L1 well developed, apically truncate, with one deep lateral notch; L2 and L3 represented by small unsclerotized points. ***Paraphyses***: Interlobular spaces between L1 and L2 and between L2 and L3 each with 2 clavate paraphyses; first pair similar in length to L1 and second pair nearly 1/2 that length. ***Plates***: All plates rather simple, roughly triangular in shape with minimal fringing, and shorter in length than L1. One, minute, simple pair between L1; 2 present between L1 and L2, each with 1–2 short lateral fringes; 3 present between L2 and L3, simple or with one lateral fringe; plates absent anterior of seta marking position of L3. ***Ducts***: Dorsal pygidial macroducts of uniform size; 1 marginal macroduct present between median lobes, 30–37 (median 34) μm in length, surpassing posterior margin of anal opening; 3–5 (median 3.5) macroducts arising from space between L1 and L2 (abdominal segment VII), 10–16 (median 14) on abdominal segment VI, 13–24 (median 17) on abdominal segment V, with a total of 31–42 (median 34) dorsal macroducts on each side of pygidium. Clusters of pre-pygidial macroducts present on dorsal submargins, 11–17 (median 13) on each side of abdominal segment IV, 10–16 (median 12) on segment III, 8–11 (median 10) on segment II, fewer present up to mesothorax. Ventral microducts few; present on head and thorax in submarginal and submedial rows; present in submargins of abdominal segments I–VI. ***Anal opening***: Oval, maximum diameter (length) 14–23 (median 20) μm, situated 20–31 (median 25) μm, approximately 3 anal lengths, anterior to base of L1. ***Perivulvarpores***: Absent.

**Figure 5.**
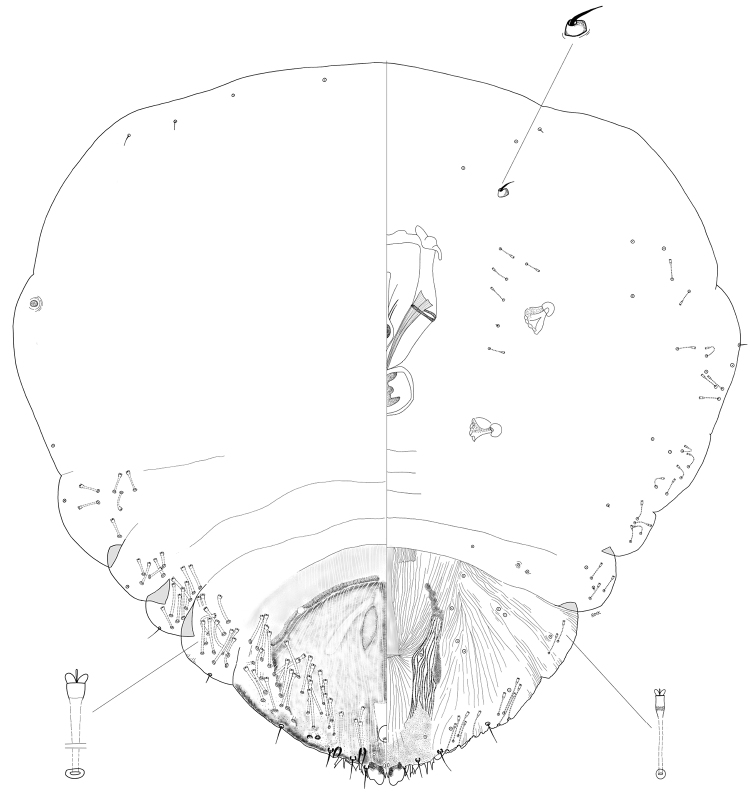
*Hemiberlesiaozolita* sp. nov. Adult female, full body view, illustrated from a paratype (D0288C).

#### DNA sequences.

DNA sequences of several loci of *Hemiberlesiaozolita* sp. nov. have been published from one paratype (D0288C) and one other individual from the type series that was ground to powder during the preparation of DNA (D0288A): 28S, GenBank accession numbers MH933989.1 (D0288C) and KY218997.1 (D0288A); EF-1α, MH915719.1 (D0288C) and KY221290.1 (D0288A); COI-II, MH916225.1 and MH916397.1 (D0288C), GQ425001.1 (D0288A); 16S of primary endosymbiont (*Uzinuradiaspidicola*), KY220099.1.

**Figure 6.**
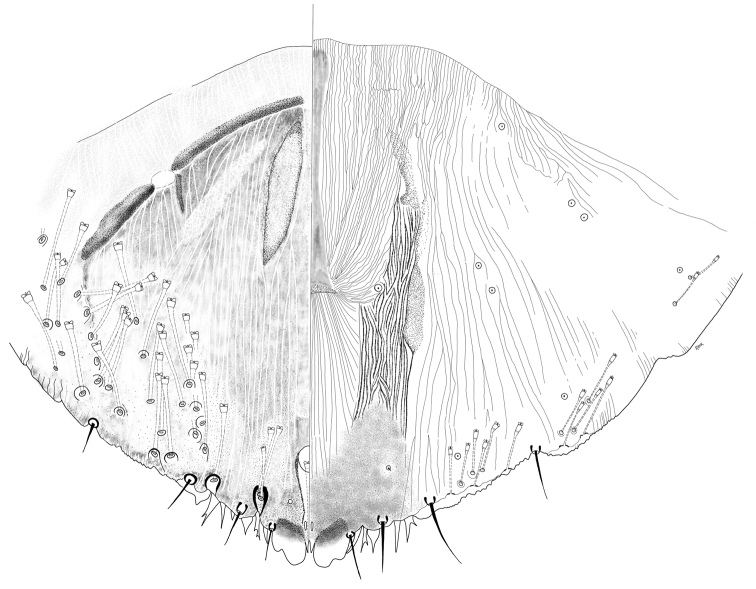
*Hemiberlesiaozolita* sp. nov. Adult female, expanded view of pygidium, illustrated from a paratype (D0288C).

#### Informal synonyms.

Specimens from the type series and their endosymbionts have appeared in several published phylogenetic trees, and have been referred to variously as “Diaspidiotus sp undesc #1” (Morse and Normark 2006; Rugman-Jones et al. 2010), “Diaspidiotus sp nov 1” (Andersen et al. 2010), and “Hemiberlesia ud0288” (Schneider et al. 2018; Normark et al. 2019).

#### Remarks.

*Hemiberlesiaozolita* sp. nov. is most similar to *H.nothofagi* Williams, but *H.ozolita* sp. nov. is distinctive in having plates in the first space shorter than L1, 3 plates beyond L2, anal opening relatively small (< 25 μm in diameter), 31–42 macroducts on each side of the pygidium, and groups of ventral submarginal microducts running from the pygidium to the thorax. In contrast, *H.nothofagi* has plates in the first space exceeding L1 in length, 6–7 plates beyond L2, a large anal opening (30 μm in diameter), about 26 pygidial macroducts per side, and few microducts present on the venter, not arranged in submarginal groups on thoracic and pre-pygidial segments. The new species is also similar to *H.rapax* except it has a much smaller anal opening and the pre-pygidial macroducts are longer, about as long as the pygidial macroducts.

*Hemiberlesiaozolita* sp. nov. constitutes the sister-lineage of a clade that includes all other sampled *Hemiberlesia* species and *Palinaspissordidata*, according to the phylogenetic estimate of Schneider et al. (2018). The relatively small anal opening in this species is a trait shared in common with several other species formerly placed in *Abgrallaspis* that have since been transferred to *Hemiberlesia* (Normark et al. 2014), a decision supported by molecular evidence.

#### Host plant.

Not recorded.

#### Etymology.

The specific epithet is an adjective formed from the Greek terms *ozotos*, meaning branching, and *litos*, meaning simple, and is used to describe the distinctly simple pygidial plates of this species.

#### Distribution.

Argentina (Jujuy).

### Melanaspis lilloi

Taxon classificationAnimaliaHemipteraDiaspididae

Schneider, Claps, Wei, Normark & Normark sp. nov.

832068A3-4ABC-5387-87CB-BB5D16AE81F4

http://zoobank.org/1559ADD4-4074-4033-B94B-4B1E4441A974

Figs 7 , 8

#### Material examined.

***Holotype***: Argentina • 1 adult female; Jujuy, 30 km N Humahuaca; 22.97S, 65.39W; 12.II.2002; L. E. Claps, P. Zamudio, L. Diaz-Briz, & P. Cabrera leg.; IFML, L. E. Claps catalog #5-02, #1092 (D0275L). ***Paratypes***: Argentina • 3 adult females; same data as holotype; USNM (D0275H) • 1 adult female; same data as holotype; USNM (D0275G) • 1 adult female; same data as holotype; USNM (D0275K) • 4 adult females; same data as holotype; UMEC (D0275I) • 1 adult female; same data as holotype; UMEC (D0275M) • 4 adult females; same data as holotype; IFML (D0275J) • 1 adult female; Jujuy, Humahuaca, entrada a Iruya; 22.997S, 65.356W; 12.II.2002; L. E. Claps, P. Zamudio, L. Diaz-Briz, & P. Cabrera leg.; IFML, L. E. Claps catalog #15-02 (D0297C).

**Figure 7.**
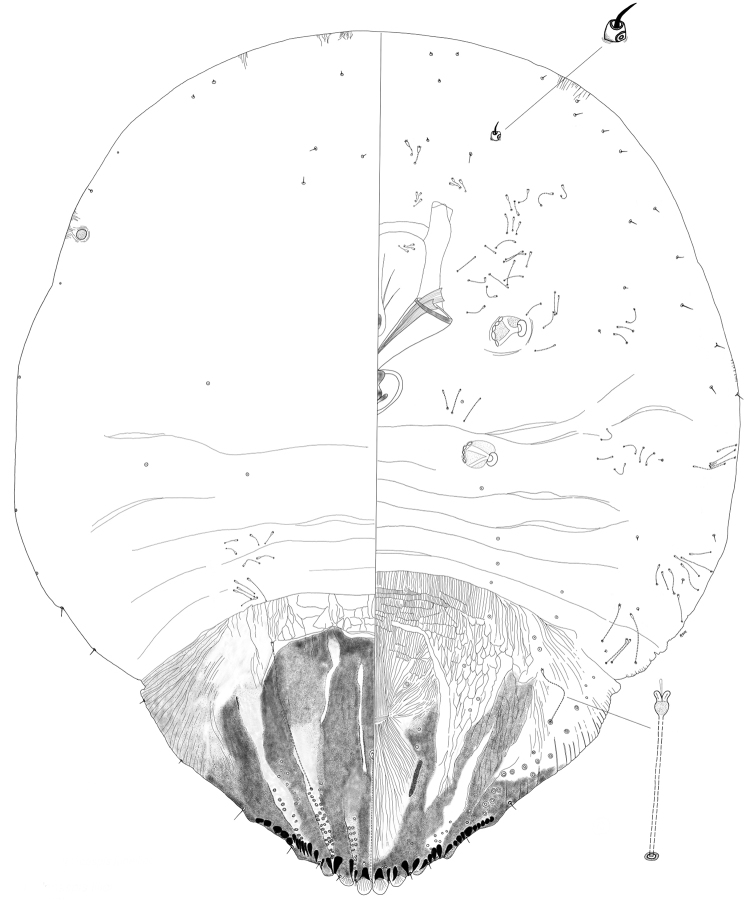
*Melanaspislilloi* sp. nov. Adult female, full body view, illustrated from the holotype (D0275L).

#### Description

(*N* = 16). Adult female presumed to secrete scale cover, not pupillarial. Appearance in life not recorded. Slide-mounted adult female 930–1610 (median 1350, holotype 1610) μm long, 820–1350 (median 1160, holotype 1330) μm wide; broadest near mesothorax. Body outline turbinate. Derm membranous throughout at maturity except for pygidium, which has characteristic dorsal sclerotized areas; sclerotization of these areas unusually heavy, such that paraphyses and basal scleroses of lobes difficult to discern clearly on some specimens. Antennae, simple, each with 1 long seta, distance between antennae 200–330 μm (median 260). Without disc pores near spiracles. ***Lobes***: With 4 pairs of well-developed pygidial lobes, L1–L3 apically rounded and L4 truncate or pointed, notches absent from lobes; L1 slightly wider than long, median lobes separated by narrow space 0.15 times width of L1, with basal sclerosis about 1/2 width of L1 arising from mesal edge; L2 and L3 similar in size and shape, shorter and broader than L1; L4 somewhat variable in shape, truncate or with sloping edges. ***Paraphyses***: Short and clavate, scarcely longer than L1; absent between L1, paraphysis formula 2-2-3 or 2-2-4; 1 interlobular paraphysis near outer corner of L1, 1 attached to inner corner of L2, 1 in interlobular space between L2 and L3, 1 attached to inner and outer corners of L3, 1 narrow paraphysis attached to inner corner of L4 and 2–3 narrow paraphyses in interlobular space between L3 and L4, these often fused into a single complex mass and difficult to count; several paraphysis-like sclerotizations surrounding macroduct orifices present beyond L4. ***Plates***: Apparently absent. ***Ducts***: Dorsal pygidial macroducts of 1-barred type, nearly uniform in size, with minute orifices and long slender ducts, most arranged in distinct furrows between sclerotized areas arising from interlobular spaces; 1 submarginal macroduct orifice immediately anterior to each L1, with ducts extending beyond posterior margin of anal opening; 10–21 (median 16) duct orifices in furrow of first space, originating between L1 and L2 and extending in elongate cluster anteriorly 60–85% of distance to anus, each duct about 120–130 μm in length; 18–40 (median 29) in furrow of second space, originating between L2 and L3 and extending anteriorly to a point laterad or anterolaterad of anus; 3–9 (median 6) on sclerotized area arising from L3; 2–24 (median 15) in furrow of third space, originating between L3 and L4 and extending anteriorly to a point anterolaterad of anus; duct orifices in furrows of second and third spaces membranous, especially towards anterolateral corner of furrow, or surrounded by partial or complete sclerotized ring, especially near posterior end and along medial margin of furrow; submedial clusters of dorsal macroducts present on each pre-pygidial abdominal segment, shorter and narrower than pygidial ducts. Ventral pygidial microducts similar to dorsal macroducts in size and shape and similarly arranged in rows on segments V–VII, 21–44 (median 33) on each side; ventral duct orifices on segment V each surrounded by conspicuous sclerotized ring, degree of sclerotization decreasing towards anterolateral corner of segment; microducts also distributed along head, thorax, and pre-pygidial margins, as well as rows extending from marginal area toward each spiracle. ***Anal opening***: Oval, 20–28 (median 24) μm long, positioned 3.5–6.3 (median 4.6) anal lengths (85–124, median 107 μm) from base of L1, near midpoint of pygidium. ***Perivulvarpores***: Absent.

**Figure 8.**
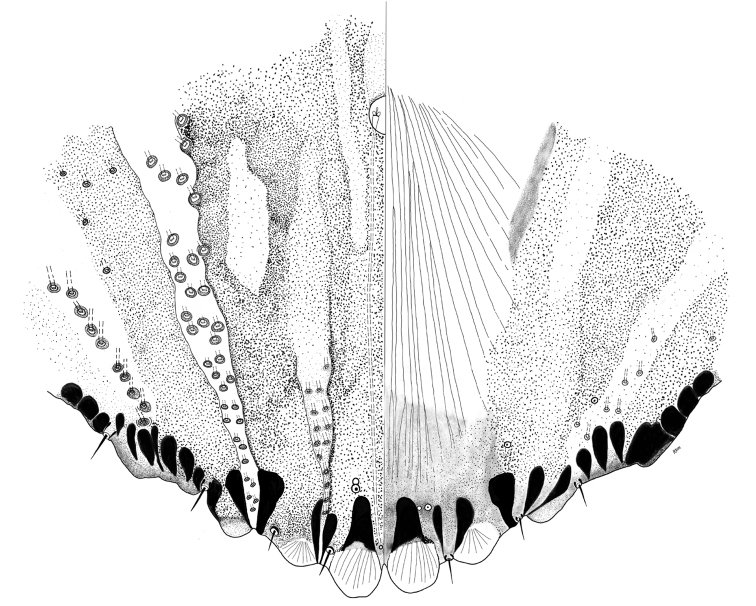
*Melanaspislilloi* sp. nov. Adult female, expanded view of pygidium, illustrated from the holotype (D0275L).

#### DNA sequences.

Several DNA sequences of *Melanaspislilloi* sp. nov. have been published, including fragments of 4 loci from a paratype (D0275G): 28S, GenBank accession number KY218989.1; EF-1α, MH915714.1 and KY221286.1; CAD, MH915988.1; and COI-II, KY22069.1 and MH916394.1. DNA sequences have also been published for other members of the type series that were ground to powder during DNA preparation; these include sequences of 28S (D0275D, DQ145363.2; D0297A, DQ145362.2 and KY219142.1), EF-1α (D0275D, DQ145475.1; D0297A, DQ145474.1 and KY221295.1), and COI-II (D0275, GQ445417.1; D0297A, GQ425005.1). DNA sequences of the primary bacterial endosymbiont, *Uzinuradiaspidicola*, of *M.lilloi* sp. nov. have also been published for ground-up individuals of the type series, including fragments of 16S rDNA (D0275, DQ133558.1 and DQ868836.1; D0297A, GQ424858.1) and 23S rDNA (D0275, DQ873248.1).

#### Informal synonyms.

Specimens from the type series have appeared in several published phylogenetic trees, and have been referred to variously as “Melanaspis sp. nov.” (Gruwell et al. 2005) “Melanaspis sp undesc #2” and “Melanaspis sp undesc #3” (Gruwell et al. 2007; Morse and Normark 2006; Rugman-Jones et al. 2010), “Melanaspis sp. undesc.” (Gruwell et al. 2009), “Melanaspis sp nov 1” and “Melanaspis sp nov 2” (Andersen et al. 2010), and “Melanaspis ud0276” (Schneider et al. 2018; Normark et al. 2019).

#### Remarks.

This species is very similar to *M.targionoides* sp. nov. The diagnosis and affinities of *M.lilloi* sp. nov. are discussed below under the remarks for *M.targionoides* sp. nov.

#### Host plant.

Not recorded.

#### Etymology.

The specific epithet is a noun in the genitive case, meaning “of Lillo”. It honors the Instituto Miguel Lillo, academic home of Lucia Claps and the other scientists who first collected the species described in this manuscript.

#### Distribution.

Argentina (Jujuy).

### Melanaspis targionoides

Taxon classificationAnimaliaHemipteraDiaspididae

Schneider, Claps, Wei, Normark & Normark sp. nov.

902F2FF5-AE8A-515F-A80E-3A9C9DF0EEA3

http://zoobank.org/2416C2B7-1A4F-40E7-BEE3-18536816FF23

Figs 9 , 10

#### Material examined.

***Holotype***: Argentina • 1 adult female; Jujuy, entre Maimará & Tilcara; 23.586S, 65.408W; 13.II.2002; L. E. Claps, P. Zamudio, L. Diaz-Briz, & P. Cabrera leg.; IFML, L. E. Claps catalog #20-02, #1092 (D0272C). ***Paratypes***: Argentina • 1 adult female; same data as holotype; UMEC (D0272E) • 1 adult female; same data as holotype; UMEC (D0272F) • 4 adult females; Jujuy, Humahuaca, camino a Aparzo; 23.20S, 65,10W; 13.II.2002; L. E. Claps, P. Zamudio, L. Diaz-Briz, & P. Cabrera leg.; USNM, L. E. Claps catalog #23-02 (D0264D) • 1 adult female; same data as previous; USNM (D0264C) • 1 adult female; same data as previous; USNM (D0264E) • 1 adult female; same data as previous; USNM (D0264F) • 1 adult female; same data as previous; USNM (D0264G) • 3 adult females; Jujuy, 30 km N Humahuaca; 22.97S, 65.39W; 12.II.2002; L. E. Claps, P. Zamudio, L. Diaz-Briz, & P. Cabrera leg.; UMEC, L. E. Claps catalog #6-02 (D0276F) • 1 adult female; same data as previous; UMEC (D0276E) • 4 adult females; Jujuy, Humahuaca, entrada a Juella; 23.525S, 65.396W; 14.II.2002; L. E. Claps, P. Zamudio, L. Diaz-Briz, & P. Cabrera leg.; IFML, L. E. Claps catalog #26-02 (D0291D) • 1 adult female; same data as previous; IFML (D0291C).

**Figure 9.**
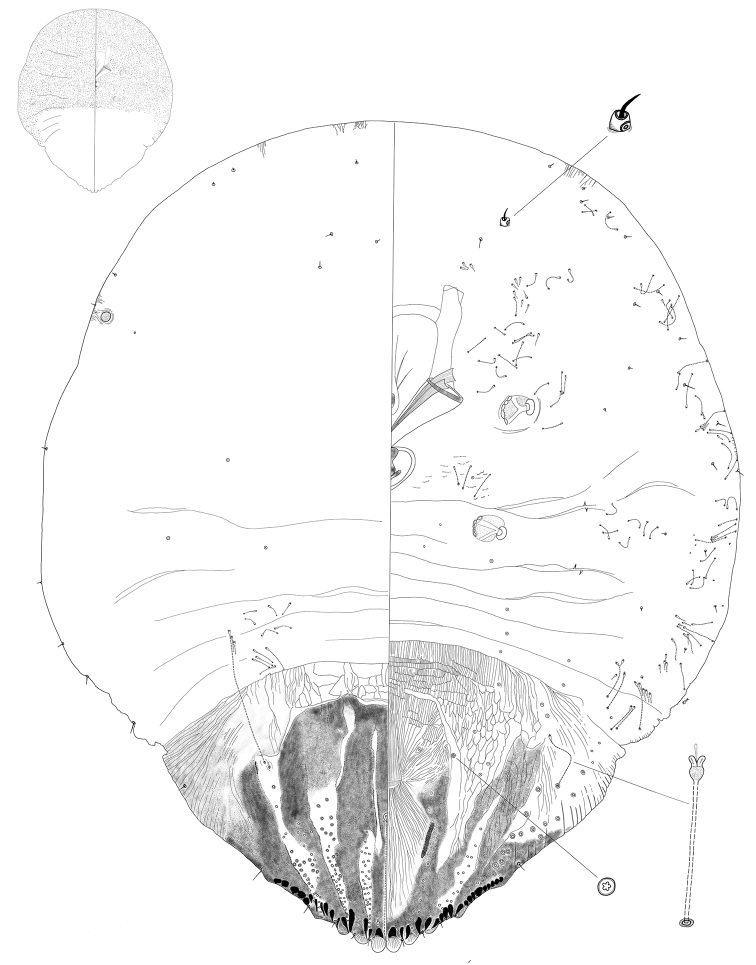
*Melanaspistargionoides* sp. nov. Teneral adult female, full body view, illustrated from the holotype (D0272C). Inset at upper left shows sclerotization pattern of a fully mature female, illustrated from a paratype (D0264E).

#### Description

(*N* = 20). Adult female presumed to secrete scale cover, not pupillarial. Appearance in life not recorded. Slide-mounted adult female 860–1720 (median 1130, holotype 1020) μm long, 730–1440 (median 960, holotype 860) μm wide; broadest near mesothorax. Body outline turbinate. Prosoma becoming sclerotized at full maturity (length > 1400 μm); derm otherwise membranous except for pygidium, which has characteristic dorsal sclerotized areas; sclerotization of these areas unusually heavy, such that paraphyses and basal scleroses of lobes difficult to discern clearly on some specimens. Antennae simple, each with 1 long seta, distance between antennae 130–410 (median 230) μm. Without disc pores near spiracles. ***Lobes***: With 4 pairs of well-developed pygidial lobes, L1–L3 apically rounded and L4 truncate or pointed, notches absent from lobes; L1 slightly wider than long, median lobes separated by narrow space 0.15 times width of L1, with basal sclerosis about 1/2 width of L1 arising from mesal edge; L2 and L3 similar in size and shape, shorter and broader than L1; L4 somewhat variable in shape, truncate or with sloping edges. ***Paraphyses***: Short and clavate, scarcely longer than L1; absent between L1, paraphysis formula 2-2-3 or 2-2-4; 1 interlobular paraphysis near outer corner of L1, 1 attached to inner corner of L2, 1 in interlobular space between L2 and L3, 1 attached to inner and outer corners of L3, 1 narrow paraphysis attached to inner corner of L4 and 2–3 narrow paraphyses in interlobular space between L3 and L4, these often fused into a single complex mass and difficult to count; several paraphysis-like sclerotizations surrounding macroduct orifices present beyond L4. ***Plates***: Apparently absent. ***Ducts***: Dorsal pygidial macroducts of 1-barred type, nearly uniform in size, with minute orifices and long slender ducts, most arranged in distinct furrows between sclerotized areas arising from interlobular spaces; 1 submarginal macroduct orifice immediately anterior to each L1, with ducts extending beyond posterior margin of anal opening; 17–36 (median 29) duct orifices in furrow of first space, originating between L1 and L2 and extending in elongate cluster anteriorly 90% of distance to anus or farther, anterior end of cluster usually directly laterad of anus, each duct about 120–130 μm in length; 18–53 (median 35) in furrow of second space, originating between L2 and L3 and extending anteriorly to a point laterad or anterolaterad of anus; 7–14 (median 9) on sclerotized area arising from L3; 15–30 (median 19) in furrow of third space, originating between L3 and L4 and extending anteriorly to a point anterolaterad of anus; duct orifices in furrows of second and third spaces each surrounded by sclerotized ring; submedial clusters of dorsal macroducts present on each pre-pygidial abdominal segment, shorter and narrower than pygidial ducts. Ventral pygidial microducts similar to dorsal macroducts in size and shape and similarly arranged in rows on segments V–VII, 23–51 (median 35) on each side; ventral duct orifices on segment V each surrounded by conspicuous sclerotized ring, degree of sclerotization decreasing towards anterolateral corner of segment; microducts also distributed along head, thorax, and pre-pygidial margins, as well as rows extending from marginal area toward each spiracle. ***Anal opening***: Oval, 14–31 (median 20) μm long, positioned 4–9 (median 6) anal lengths (102–144 μm, median 129 μm) from base of L1, near midpoint of pygidium. ***Perivulvarpores***: Absent or present; 0–11 (median 0, holotype 1) pores in total, distributed as one loose cluster on only one side of the body (5 of 7 individuals with pores present) or as one loose cluster on each side of the body.

**Figure 10.**
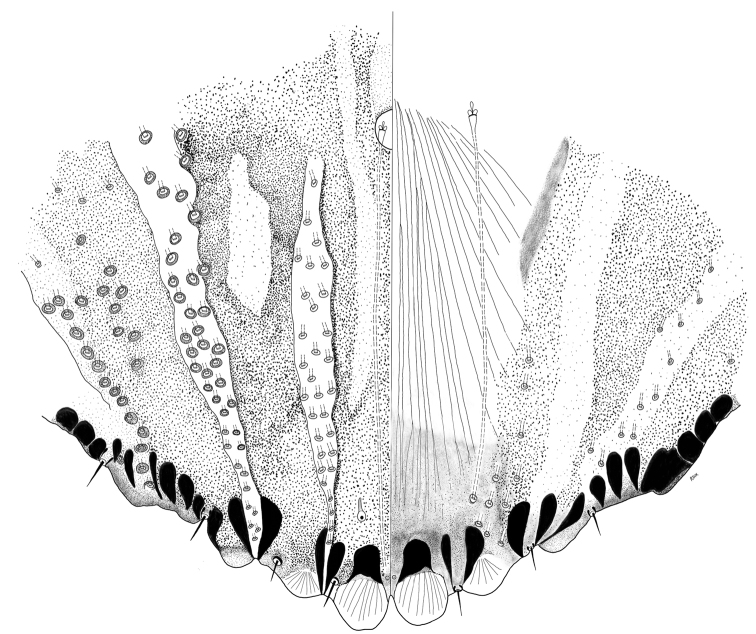
*Melanaspistargionoides* sp. nov. Adult female, expanded view of pygidium, illustrated from the holotype (D0272C).

#### DNA sequences.

Several DNA sequences of *Melanaspistargionoides* sp. nov. have been published, including fragments of 3 loci from the holotype (D0272C): large ribosomal subunit (28S, GenBank accession number KY218986.1), elongation factor 1-alpha (EF-1α, MH915711.1), and carbamoylphosphate synthetase (CAD, MH915986.1). DNA sequences have also been published for the paratypes D0276E (28S, KY218990.1; EF-1α, MH915715.1; CAD, MH915989.1) and D0264C (28S, KY218983.1). Several other members of the type series were ground to powder during DNA preparation; morphological vouchers were not preserved, but DNA sequences were published. These include sequences of 28S (D0264A, DQ145361.2; D0264C, KY218983.1; D0291A, KY219004.1 and DQ145395.2), EF-1α (D0264A, DQ145473.1), and cytochrome oxidase I and II (COI-II, D0276G, MH919222.2). An additional sequence of COI-II purporting to be from a member of the type series of this species, D0264A, was available on GenBank from 2010–2020 under accession number GQ424989.1. This was actually a sequence of a different species, *Aonidomytilusespinosai* Porter, GQ424988.1, erroneously assigned due to contamination or mislabeling; it has been retracted from GenBank. DNA sequences of the primary bacterial endosymbiont, *Uzinuradiaspidicola*, of *M.targionoides* sp. nov. have also been published, including a fragment of the small ribosomal subunit (16S) of a paratype (D0264C, KY220091.1) and 2 ground-up specimens of the type series (D0264A, GQ424852.1; D0291A, DQ868844.1), and a fragment of the large ribosomal subunit (23S of D0291B, DQ873256.1).

#### Informal synonyms.

Specimens from the type series and their endosymbionts have appeared in several published phylogenetic trees, and have been referred to variously as “Melanaspis sp. nov.” (Gruwell et al. 2005), “Melanaspis sp undesc #1” and “Melanaspis sp undesc #4” (Gruwell et al. 2007; Morse and Normark 2006; Rugman-Jones et al. 2010), “Melanaspis sp. undesc.” (Gruwell et al. 2009), “Melanaspis sp nov 1” (Andersen et al. 2010), and “Melanaspis ud0276” (Schneider et al. 2018; Normark et al. 2019).

#### Remarks.

This species is very similar to the previous one, *Melanaspislilloi* sp. nov. The two were considered to belong to a single undescribed species, (“*Melanaspis* ud0276”) by Schneider et al. (2018) and Normark et al. (2019). The diversity of informal designations assigned to members of the two species prior to 2018 were record-keeping artifacts and did not reflect any diversity of evidence-based hypotheses. Careful study of the type series during the preparation of this manuscript revealed slight but consistent morphological differences between what are here regarded as separate species. The two species are also distinguishable by DNA, and appear as separate clusters in published phylogenies (Morse and Normark 2006; Normark et al. 2019). The pattern is seen most clearly in figure S2 of Normark et al. (2019), and is seen in each of the loci sampled from both species in that study: 2.2% divergence at 28S (compared to 0.14% within *M.lilloi* sp. nov.), 2.2–2.3% divergence at EF-1α (compared to 0.12–0.35% within each species), and 1.45% divergence at the primary endosymbiont’s 16S locus (compared to 0.18–0.19% within each species). Although the species are morphologically very similar, *M.targionoides* sp. nov. has more numerous and more sclerotized dorsal ducts, along with sclerotization of the prosoma at full maturity. Specifically, the two species may be distinguished by the following characters. In *M.targionoides* sp. nov., the elongate cluster of dorsal ducts in the furrow of the first space (arising between L1 and L2) extends anteriorly at least 90% of the distance to the anus, its anterior end usually being directly lateral to the anus; in *M.lilloi* sp. nov., this cluster of dorsal ducts extends only 60–85% of the distance to the anus, its anterior end always lying posterolateral to the anus. In *M.targionoides* sp. nov. the furrow of the third space (arising between L3 and L4) has a single or double line of conspicuous subcircular sclerotized duct orifices extending from near the posterior margin to the anterior third of the pygidium; in *M.lilloi* sp. nov., the furrow of the third space has sclerotized duct openings only in the posterior third of the pygidium, with sometimes a few present further anteriorly along the medial edge of the furrow (lateral edge of the sclerotized area arising from L3) – these are often only partially sclerotized and anteroposteriorly compressed, thus appearing as partial ellipses rather than complete circles. *Melanaspistargionoides* sp. nov. has the prosoma sclerotized at full maturity (body length greater than 1.4 mm); *M.lilloi* sp. nov. has the prosoma membranous at full maturity. *Melanaspistargionoides* sp. nov. sometimes has perivulvar pores; *M.lilloi* sp. nov. lacks perivulvar pores.

*Melanaspistargionoides* sp. nov. and *M.lilloi* sp. nov. are referrable to *Melanaspis* based upon the characteristic sclerotization pattern of the dorsal pygidium, and their placement in *Melanaspis* is supported by molecular evidence (Morse and Normark 2006; Andersen et al. 2010; Rugman-Jones et al. 2010; Schneider et al. 2018; Normark et al. 2019;) . However, they possess a combination of traits often seen in species of *Targionia*, including the absence of plates, presence of numerous small, slender macroducts arranged in distinct furrows, and simple, rounded pygidial lobes lacking notches. Plates are typically present in species of *Melanaspis* but can be highly reduced and difficult to view. The simple lobes and short paraphyses found in *M.targionoides* sp. nov. and *M.lilloi* sp. nov. are similar in appearance to those of *M.enceliae* (Ferris), but the number and distribution of macroducts is quite distinct from any other species observed for this genus.

#### Host plant.

Not recorded.

#### Etymology.

The specific epithet is an adjective describing the resemblance this species bears to others placed in *Targionia* by adding the suffix -*oides* to indicate likeness in form.

#### Distribution.

Argentina (Jujuy).

### Key to species of Aspidiotini in Argentina based on adult females

**Table:** 

1	Paraphyses or paraphysis-like sclerotizations absent	**2**
–	Paraphyses or paraphysis-like sclerotizations present	**4**
2	With two pairs of well-developed pygidial lobes, L3 entirely absent; perivulvar pores absent; margin of prosoma sclerotized	***Chortinaspisjujuyensis* sp. nov.**
–	With three pairs of well-developed pygidial lobes, L3 at least represented by narrow processes; perivulvar pores present; margin of prosoma membranous	**3**
3	Anal opening about same size in diameter as length of L1, or longer; dorsal setae associated with outer corners of L2 and L3 slender, not thickened basally	***Aspidiotusnerii* (Bouché)**
–	Anal opening noticeably shorter in diameter than length of L1; dorsal setae associated with outer corners of L2 and L3 thickened, swollen basally	***Oceanaspidiotusspinosus* (Comstock)**
4	Paraphyses or paraphysis-like sclerotizations present anterior to position of L3	**5**
–	Paraphyses or paraphysis-like sclerotizations absent anterior to position of L3	**19**
5	Dorsum of pygidium with several sclerotized areas divided by furrows, one of which isolates L2 from remaining sclerotized areas; anterior submarginal region of dorsal pygidium appears reticulated or striped with variously branching furrows; longest paraphyses attached to lobes, never arising from interlobular spaces (*Crenulaspidiotus*)	**6**
–	Pygidium not as described above, L2 not isolated on a distinct sclerotized area; dorsal submargins of pygidium without reticulated or branched furrows, region patterned by fine parallel cuticular lines running perpendicular to body margin; longest paraphyses often arising from interlobular spaces	**9**
6	Interlobular paraphysis formula in first, second, and third spaces 1-1-1	***Crenulaspidiotusmaurellae* (Laing)**
–	Interlobular paraphysis formula in first, second, and third spaces, 1-2-2	**7**
7	Ventral microducts present in area anterior to interlobular space between L3 and L4; dorsal submargin adjacent to L4 reticulate	***Crenulaspidiotuscyrtus* Miller & Davidson**
–	Ventral microducts present in area anterior to interlobular space between L3 and L4; dorsal submargin adjacent to L4 striped	**8**
8	Segments I and II with conspicuous pre-pygidial lobes	***Crenulaspidiotusgreeneri* Miller & Davidson**
–	Segments I and II without conspicuous pre-pygidial lobes	***Crenulaspidiotuslahillei* (Lizer & Trelles)**
9	Body elongate, length approximately 2X maximum width	***Pseudischnaspisbowreyi* (Cockerell)**
–	Body round or turbinate, length less than 2X maximum width	**10**
10	Pygidium elongate and apically acute, with wide base and straight or concave sides tapering to point; angle formed by lateral pygidial margins being less than 90 degrees (*Acutaspis*)	**11**
–	Pygidium short and apically broad, margins usually convex; pygidial angle usually greater than 90 degrees	**14**
11	Cephalic margin of body with median notch; with slight indication of prothoracic tubercles; paraphysis arising from outer angle of L2 longer than any others arising between L2 and L3; paraphysis arising from outer angle of L3 among longest representatives	***Acutaspisreniformis* (Cockerell)**
–	Cephalic margin of body without median notch; without any indication of prothoracic tubercles; paraphysis arising from outer angle of L2 shorter than at least one other paraphysis arising between L2 and L3; paraphysis arising from outer angle of L3 noticeably shorter than longest representatives	**12**
12	Derm sclerotized around body margins, leaving central portion membranous; adult female body can reach over 2 mm in length	***Acutaspisscutiformis* (Cockerell)**
–	Derm membranous throughout except for pygidium; adult female body typically less than 2 mm in length	**13**
13	Perivulvarpores arranged in 4 small but distinct groups; longest paraphysis arising from interlobular space	***Acutaspispaulista* (Hempel)**
–	Perivulvarpores in lateral groups alone, forming an irregular longitudinal series of pores; longest paraphysis arising from lateral angle of lobe	***Acutaspisaliena* (Newstead)**
14	Plates well developed and fimbriate; dorsal macroduct orifices vary in size	***Lindingaspisrossi* (Maskell)**
–	Plates simple, minimally fringed, or absent; dorsal macroduct orifices of uniform size	**15**
15	Plates present; cephalic margin with sclerotized protuberance (*Mycetaspis*)	**16**
–	Plates absent; cephalic margin without sclerotized protuberance	**17**
16	Median lobes each with broad basal sclerosis as wide as L1 at proximal base	***Mycetaspispersonata* (Comstock)**
–	Median lobes each with narrow basal sclerosis arising only from medial angle of lobe	***Mycetaspisapicata* (Newstead)**
17	Dorsum of pygidium with heavily sclerotized areas separated by lightly sclerotized pore furrows	**18**
–	Dorsum of pygidium without heavily sclerotized areas between pore furrows	***Targioniafabianae* Leonardi (in part)**
18	Furrow of third space with continuous single or double row of conspicuous sclerotized duct openings extending from submargin to anterior third of pygidium; furrow of first space usually with more than 20 duct orifices, extending from submargin at least 90% of distance to anus; prosoma sclerotized at full maturity (when body length > 1.4 mm); with or without perivulvar pores	***Melanaspistargionoides* sp. nov.**
–	Furrow of third space without continuous row of sclerotized duct openings extending from margin to anterior, sclerotized duct openings occurring only in posterior third of furrow and sporadically along the medial margin; furrow of first space usually with fewer than 20 duct orifices, extending from submargin only 60–85% of distance to anus; prosoma remaining membranous; without perivulvar pores	***Melanaspislilloi* sp. nov.**
19	Pygidium with 3 definite pairs of sclerotized lobes, each similar in shape and size	**20**
–	Pygidium with at most 2 definite pairs of sclerotized lobes of similar shape, if present L3 represented by small sclerotized point	**24**
20	Paraphyses often obscure and shorter than L1; prosoma of mature adult female heavily sclerotized; body reniform in shape (*Aonidiella*)	**21**
–	Paraphyses obvious and longer than L1; prosoma of mature adult female membranous; body turbinate in shape (*Chrysomphalus*)	**23**
21	With 24–38 dorsal macroducts on each side of pygidium; without apophyses or scleroses anterolaterad of vulva	***Aonidiellataxus* Leonardi**
–	With 20–26 dorsal macroducts on each side of pygidium; with apophyses or scleroses anterolaterad of vulva	**22**
22	Normally each apophysis anterolaterad of vulva with 2 associated scleroses; paraphysis formula of 3-3-1 or 3-2-2	***Aonidiellaaurantii* (Maskell)**
–	Normally each apophysis anterolaterad of vulva without adjacent scleroses; rarely with faint scleroses; paraphysis formula of 2-2-0, 2-3-0, 3-3-0, 3-3-1 or 3-2-2	***Aonidiellacitrina* (Coquillett)**
23	Dorsal macroducts in second and third furrows few, arranged in single rows; pre-pygidial abdominal segment II lacking a dorsal cluster of 4 or more ducts	***Chrysomphalusdictyospermi* (Morgan)**
–	Dorsal macroducts in second and third furrows more numerous, arranged in double or triple rows; pre-pygidial abdominal segment II with a cluster of 4 or more dorsal ducts	***Chrysomphaluspinnulifer* (Maskell)**
24	Pygidial plates absent	***Targioniafabianae* Leonardi (in part)**
–	Pygidial plates present	**25**
25	Distinctive plates between positions of L3 and L4, with 1 or 2 lateral tines around a central duct projecting from the body margin	***Comstockaspisperniciosa* (Comstock)**
–	Plates between positions of L3 and L4 absent, simple or fringed, without a protruding central microduct	**26**
26	Distance between posterior margin of anus and apex of L1 within about 2X longest anal diameter	**27**
–	Distance between posterior margin of anus and apex of L1 about or exceeding 3X longest anal diameter	**33**
27	Perivulvarpores absent	**28**
–	Perivulvarpores present	**31**
28	L1 and L2 each with 1 lateral notch	***Hemiberlesiadiffinis* (Newstead)**
–	L1 with 1 medial and 1 lateral notch, L2 without notches	**29**
29	L2 with rounded apex, similar in shape to L1	***Hemiberlesiacorporifusca* (Chiesa Molinari)**
–	L2 pointed, distinctly different in shape	**30**
30	L2 represented by unsclerotized point; L3 entirely absent, represented at most by rounded projection; anal opening fairly large but located at least 1X anal diameter from apex of L1	***Hemiberlesianothofagi* Williams**
–	L2 and L3 both represented by small sclerotized points; anal opening very large and located less than 1X anal diameter from apex of L1	***Hemiberlesiarapax* (Comstock)**
31	L2 and L3 represented at most by small unsclerotized points; plates beyond L3 simple, minimally fringed	***Hemiberlesialataniae* (Signoret)**
–	L2 with medial and lateral notches, L3 pointed and sclerotized; plates beyond L3 well developed and fringed	**32**
32	L2 unsclerotized, often blending in with surrounding plates; all plates highly fringed and exceeding L1 in length	***Hemiberlesiapalmae* (Cockerell)**
–	L2 sclerotized, easily distinguishable from plates; some plates minimally fringed and approximately same length as L1	***Hemiberlesiacyanophylli* (Signoret)**
33	L3 well developed; anal opening about equal in size to 1 median lobe	**34**
–	L3 represented at most by slight projection or unsclerotized point; anal opening clearly smaller than 1 median lobe	**35**
34	L1 with basal sclerosis; anal opening longer than wide; paraphyses between L1 and L2 represented by doubled pairs, forming small furrow with 4–6 macroducts	***Hemiberlesialatastei* (Cockerell)**
–	L1 without basal sclerosis; anal opening wider than long; paraphyses between L1 and L2 represented by single pair, with 2 macroducts arising from interlobular space	***Hemiberlesiamendax* McKenzie**
35	Perivulvarpores absent	**36**
–	Perivulvarpores present	**38**
36	Paraphyses arising from outer angle of L1 without swollen knob at anterior end, about equal in length to L1	***Hemiberlesiaozolita* sp. nov.**
–	Paraphyses arising from outer angle of L1 with swollen knob at anterior end, noticeably longer than length of L1	**37**
37	Paraphysis arising from outer angle of L1 mushroom-shaped in appearance; plates fringed and at least as long as L1	***Clavaspisherculeana* (Cockerell & Hadden)**
–	Paraphysis arising from outer angle of L1 swollen at anterior end but not mushroom-shaped; plates all simple and shorter in length than L1	***Clavaspissubsimilis* (Cockerell)**
38	L2 well developed or represented by sclerotized projection of pygidial margin	**39**
–	L2 represented at most by unsclerotized point	**40**
39	Posterior apex of L2 in line with or posterior to apex of L1; plates well developed, as long as L1	***Diaspidiotusancylus* (Putnam) (leaf form)**
–	Posterior apex of L2 anterior to apex of L1; plates poorly developed, shorter in length than L1	***Diaspidiotusostreaeformis* (Curtis)**
40	Minimally fringed plates present between L1–L2 and L2–L3, absent or present anterior to position of L3, broad-based with 2 or 3 fringes when present; with 1 or 2 submarginal macroducts on dorsum of IV	***Clavaspispatagonensis* sp. nov.**
–	Well fringed plates present between L1–L2 and L2–L3, only simple plates present anterior to position of L3; without submarginal macroducts on dorsum of IV	**41**
41	L1 without medial notch, with 1 lateral notch; with 2 simple plates between median lobes; 30 or more perivulvar pores present	***Diaspidiotusancylus* (Putnam) (bark form)**
–	L1 with 1 medial and 1 lateral notch; without plates between median lobes; fewer than 30 perivulvar pores present	***Diaspidiotusuvae* (Comstock)**

Additional online resources aiding in the identification of Aspidiotini are provided by Schneider et al. (2019) and Dooley (2006).

## Supplementary Material

XML Treatment for Chortinaspis jujuyensis

XML Treatment for Clavaspis patagonensis

XML Treatment for Hemiberlesia ozolita

XML Treatment for Melanaspis lilloi

XML Treatment for Melanaspis targionoides
